# Supplementary material for: Novel Ultrasound-Guided Cervical Intervertebral Disc Injection of Platelet-Rich Plasma for Cervicodiscogenic Pain: A Case Report and Technical Note
Source: Healthcare (Basel). 2022 Jul 29;10(8):1427. doi: 10.3390/healthcare10081427 (PMC9408075; doi:10.3390/healthcare10081427)
Supplement: Supplementary file 1 [file healthcare-10-01427-s001.zip › healthcare-1845723-supplementary.pdf]

Supplementary Material

Video S1.

https://www.dropbox.com/s/duuv4xmj92i5bn6/US%20guided%20Cervical%20discs%20palpation%20with%20explanation_1.mp4?dl=0

Video S2.

https://www.dropbox.com/s/6ir70f03eww23c9/Newe%20C%20spine%20disc%20injection%20with%20explanation%20and%20coloured%20pictures%20and%20label.mp4?dl=0

Video S3

https://www.dropbox.com/s/kfoci7x2by0s4m1/WH%27s%20video%20final%20edited%20with%20label%20and%20pcitures.mp4?dl=0

Video S4

https://www.dropbox.com/s/bh0es7hmurcz2yc/New%20Cervical%20disc%20injection%20using%20micro convex%20transducer_with%20explanation.mp4?dl=0
